# Interface fluid syndrome caused by the corneal perforation injury after small incision lenticule extraction: a case report

**DOI:** 10.1186/s12886-024-03339-3

**Published:** 2024-03-13

**Authors:** Xu Jing, Li Congxin, Zhang Xiaoyan, Yuan Yue, Li Jiao, Zu Peipei, Wang Yirong, Wen Ying, Bi Hongsheng

**Affiliations:** 1 Affiliated Eye Hospital of Shandong University of Traditional Chinese Medicine (TCM), No. 48#, Yingxiongshan Road, 250002 Jinan, P. R. China; 2 Shandong Provincial Key Laboratory of Integrated Traditional Chinese and Western Medicine for Prevention and Therapy of Ocular Diseases, Shandong Academy of Eye Disease Prevention and Therapy, No. 48#, Yingxiongshan Road, 250002 Jinan, P. R. China

**Keywords:** Interface fluid syndrome, Small incision lenticule extraction, Corneal perforation

## Abstract

**Background:**

To report a case of interface fluid syndrome (IFS) following traumatic corneal perforation repair after small incision lenticule extraction (SMILE).

**Case presentation:**

A 23-year-old woman, with a past history of SMILE, was struck in the left eye with a barbecue prod and subsequently underwent corneal perforation repair at local hospital. Primary wound repaired with a single 10 − 0 nylon suture at the area of leakage. After the surgery, her best corrected visual acuity (BCVA) was 20/30. Four days later, she presented at our hospital with blurred vision, and interface fluid syndrome (IFS) was diagnosed. Intraoperative optical coherence tomography (iOCT) was used to guide the resuturing of the corneal perforation in the left eye, followed by anterior chamber gas injection. At the first postoperative month, the BCVA was 20/25. The corneal cap adhered closely to the stroma, the surface became smooth.

**Conclusions:**

This case illustrates that any corneal perforation following lamellar surgery, including SMILE, may lead to IFS. It is crucial to consider the depth of corneal perforation, and intraoperative optical coherence tomography (iOCT) plays a unique role in the repair procedure.

## Introduction

Originally Interface Fluid Syndrome (IFS) was described as diffuse fluid accumulation within the flap interface after laser-assisted in-situ keratomileusis (LASIK), and following the elegant experiments by Dawson et al., and contributions by other researchers, our understanding of IFS has steadily grown [1, 2]. It is now clear that any condition leading to corneal edema may result in IFS, and also that it can present following other lamellar procedures, like SMILE [2–9]. When it manifests as an early event after LASIK, it is often associated with increased intraocular pressure (IOP) in steroid responders [2]. If it occurs as a late event, it may also be linked to elevated IOP, but more commonly, it is associated with other causes of corneal edema, such as corneal endothelial cell decompensation [3–5].

In comparison to LASIK, small incision lenticule extraction (SMILE), a more recent lamellar refractive surgery technique which involves a much smaller peripheral epithelial incision (around 2-mm in length) eliminates certain flap-related complications associated with LASIK, like flap dislocation or folds. However, it’s important to note that the potential space between the corneal cap and stromal bed still exists in SMILE, and, as mentioned, IFS has been reported following SMILE. In this report, we present a new case of IFS following suturing of a traumatic corneal perforation in a patient with past history of SMILE.

## Case presentation

**Fig. 1:**
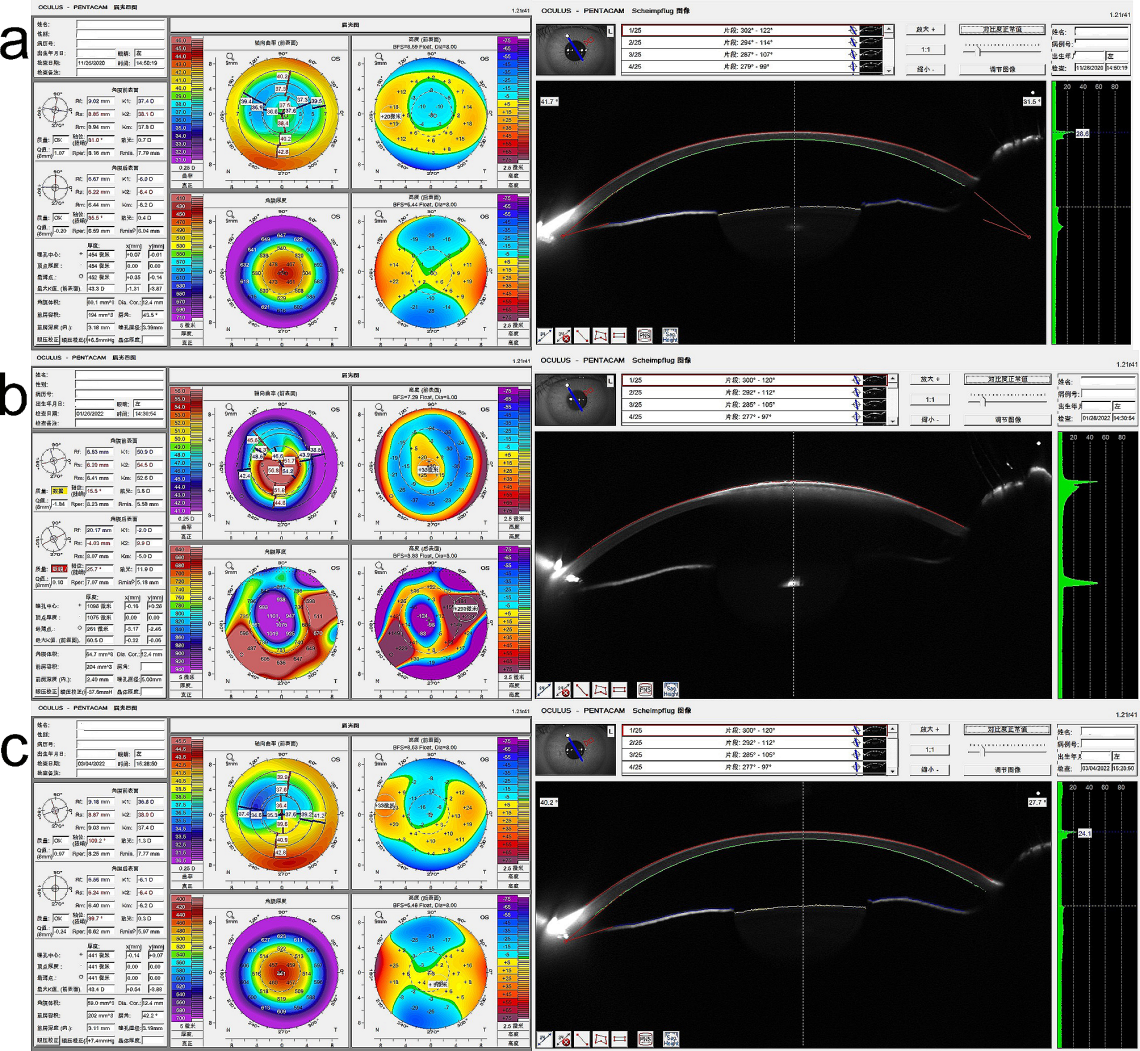
**a**: The cornea tomography after SMILE, cornea thickness was 452 μm, K1 was 37.4 D, K2 was 38.1 D. **b**: Cornea thickness was 1075 μm, K1 was 50.9 D, K2 was 54.5 D. **c**: The first postoperative month, cornea thickness was 441 μm, K1 was 36.8 D, K2 was 38.0 D

A 23-year-old woman suffered a blow to her left eye with a barbecue prod and subsequently underwent repair of corneal perforation at a local hospital. After the surgery, her best corrected visual acuity (BCVA) was 20/30. Four days later, she presented with blurred vision in her left eye and sought treatment at our hospital. Her ocular history revealed that she had undergone bilateral SMILE for myopia fifteen months earlier, with an uneventful postoperative course and 20/20 uncorrected visual acuity (UCVA) in each eye. The cornea tomography after SMILE showed that cornea thickness was 452 μm, K1 was 37.4 D, K2 was 38.1 D (Fig. 1a).

**Fig. 2:**
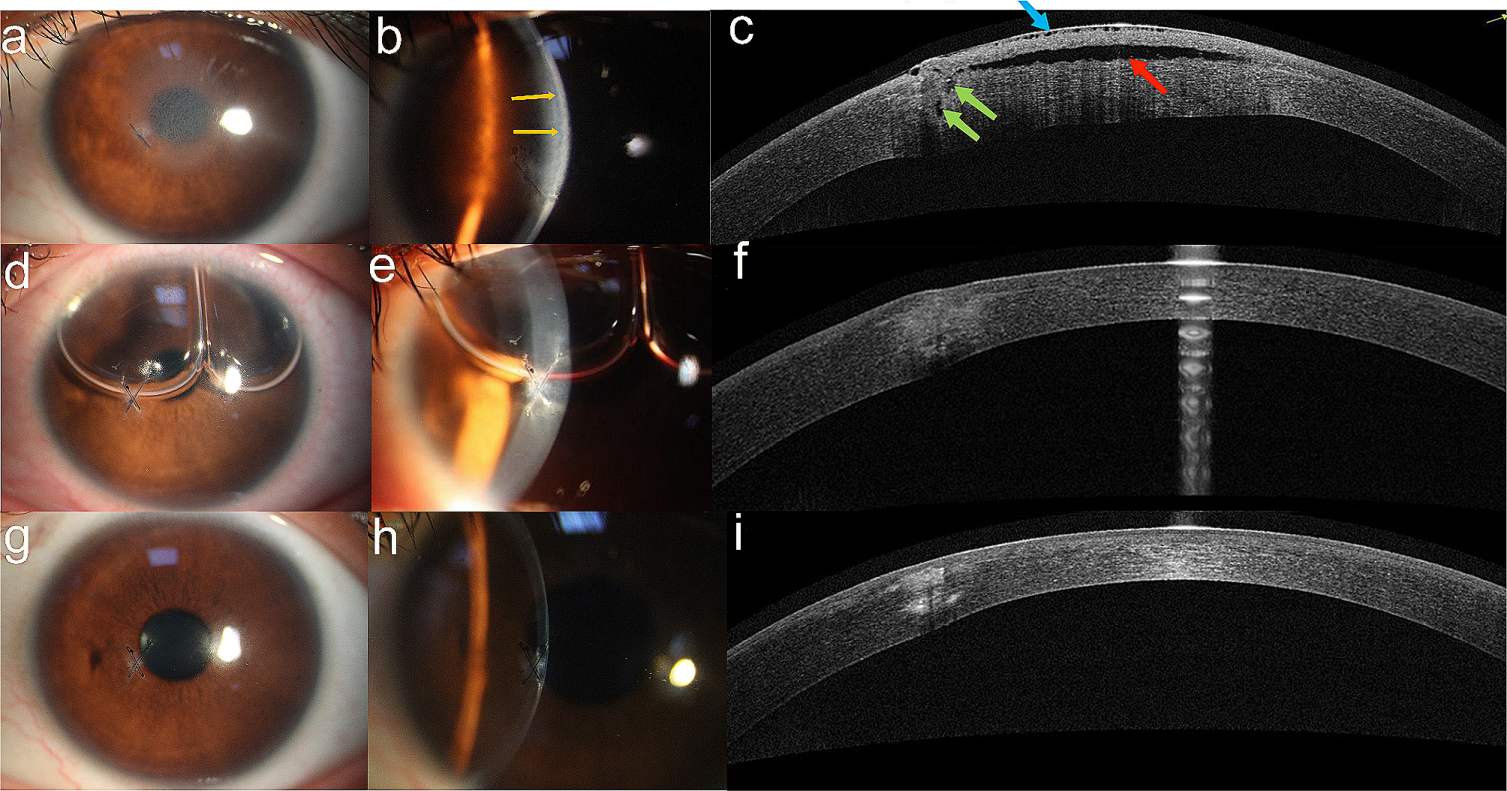
**a**, **b**: One suture can be seen under the inferonasal margin of the pupil without loosening (which was repaired at local hospital). The cornea was edematous with fluid accumulation in the lamellar interface in the eye (yellow arrows). **c**: AS-OCT showed the wound was not completely healed and communicated with the interface fluid accumulation (green arrows), the cornea had diffuse microcystic epithelial edema (blue arrow), a hyporreflection space between the cornea flap and the stromal bed was present (red arrow). **d**, **e**: Two hours postoperatively, corneal edema subsided significantly, microcystic epithelial edema and lamellar interface fluid disappear, the perforation was in good alignment. **f**: AS-OCT showed that the corneal stroma was still edema but there was no significant interface fluid. **g**, **h**: One month postoperatively, the cornea was transparent, the tissue infiltration and edema at the perforation port were further limited, the corneal surface was regular. **i**: The wound healed well and the corneal edema subsided, leaving an area of hyperreflection

Upon examination, the BCVA in her left eye was FC/40 cm. Intraocular pressure (IOP) measured by tonometry (Icare Finland Oy; TA03) was 10.2 mmHg on the central cornea and 13 mmHg peripheral to the cap. Slit-lamp examination revealed diffuse edema in the central cornea (about 5 mm in diameter). The focal area showed significant microcystic epithelial edema and tissue infiltration at the site of the penetrating wound. A fluid gap was observed at the stromal interface, extending under the central corneal cap. Primary wound repair of the corneal laceration had been done in the local hospital, with a single 10 − 0 nylon suture at the area of leakage (Fig. 2a, b).

**Fig. 3:**
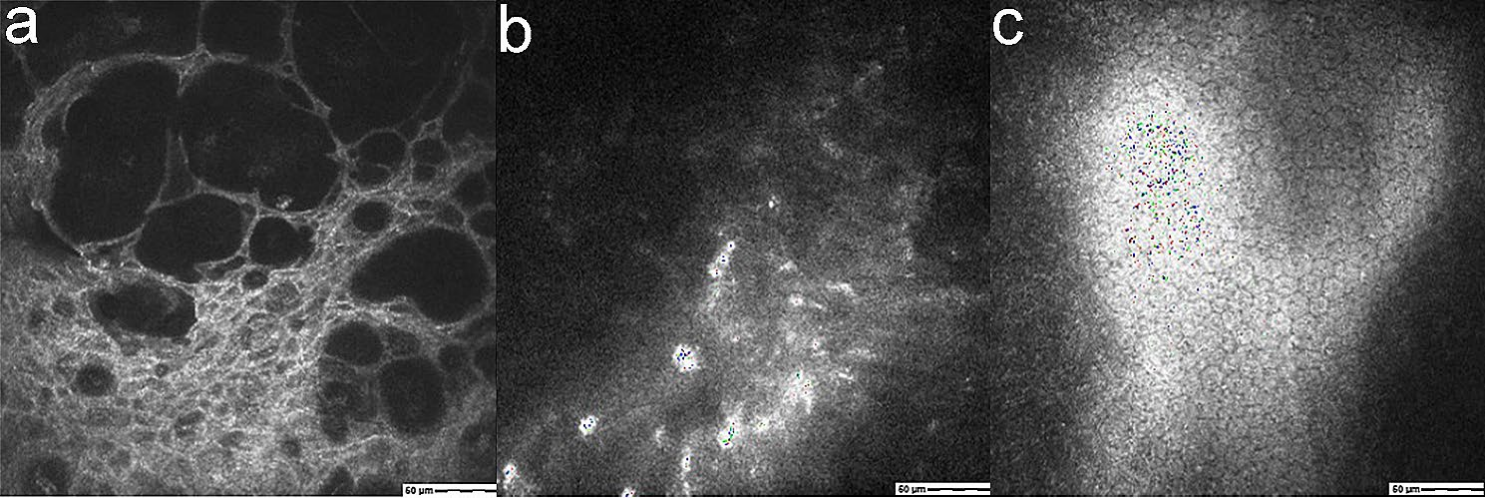
**a**: Confocal microscopy revealed edema and thickening of the corneal epithelial tissue at the lesion site, along with numerous hyporeflective areas. **b**: Corneal stroma structure was irregular. Inflammatory cells, as well as other pathogenic bacteria, were not observed. **c**: The endothelial cells appeared uniform, with a density of 2800 cells/mm^2^

Confocal microscopy revealed edema and thickening of the corneal epithelial tissue at the lesion site, along with numerous hyporeflective areas and a disordered stroma layer. The endothelial cells appeared uniform, with a density of 2800 cells/mm^2^, and no obvious inflammatory cell signals were observed (Fig. 3).

Anterior segment optical coherence tomography (AS-OCT) displayed a hyporeflective space between the corneal cap and stromal bed. The inner opening of the wound was cracked, and the wound path was poorly aligned. The wound communicated with the anterior chamber and the hyporeflective space. The depth of the corneal suture was approximately half of the corneal thickness (Fig. 2c). Cornea thickness was 1075 μm, K1 was 50.9 D, K2 was 54.5 D (Fig. 1b).

To address the issue, intraoperative optical coherence tomography (iOCT) was used to guide the resuturing of the corneal perforation in the left eye and anterior chamber gas injection. After removing the previous suture, two new interrupted 10 − 0 nylon sutures were used to repair the corneal laceration in the area of leakage. The procedure was guided by iOCT to ensure suture depth up to the Descemet layer and closure of the full-thickness wound tract. Intraoperative compression from the opposite side of the wound led to a large amount of liquid drainage. The iOCT showed that the corneal stroma was still edematous, but there was no significant interface fluid. Additionally, a corneal puncture was performed at 2 o’clock with a blade to slowly release part of the aqueous humor and reduce IOP. Filtered air was injected to fill the anterior chamber, maintaining the IOP slightly higher than normal. The patient was instructed to remain in the supine position until the gas was absorbed.

Two hours after the surgery, the UCVA of the left eye improved to 20/30, and the IOP was 10.0 mmHg (NIDEK NT-510; NIDEK; JA-PAN). Slit-lamp photographs revealed significant subsiding of corneal edema, disappearance of microcystic epithelial edema and lamellar interface fluid, proper alignment of the perforation, and a normal-depth anterior chamber with a bubble (Fig. 2d, e). One week after surgery, AS-OCT showed that the corneal stroma was still edematous, but there was no significant interface fluid (Fig. 2f).

At the first postoperative month, the UCVA of the left eye was 20/25, and the BCVA was 20/25 (-0.25 × 11). The IOP was 12.0 mmHg. The corneal cap adhered closely to the stroma, hyperreflection at the penetrating wound gradually diminished, the surface became smooth, the internal wound healed well, and the hidden suture depth was approximately 3/4 of the corneal thickness (Fig. 2g, h, i). The cornea thickness was 441 μm, K1 was 36.8 D, K2 was 38.0 D (Fig. 1c).

## Discussion

Bushley et al. reported a case of IFS following LASIK caused by traumatic corneal perforation. One day after the repair of the corneal wound, the patient presented with diffuse corneal epithelial microcystic edema, lamellar interface fluid accumulation, and decreased visual acuity. They postulated that the corneal laceration was closed, but the posterior extent of the wound was longer than the anterior extent, allowing aqueous humor to diffuse through a posterior wound gape into the stroma. To address this, additional sutures were placed to close the presumed posterior wound gape [10].

Based on the examination of AS OCT, we speculate on the possible pathogenesis of the case in our study as follows: After suture treatment at the local hospital, the wound was momentarily closed, forming the anterior chamber, and leading to a visual acuity recovery to 20/30. However, due to insufficient suture depth, the entire wound canal was poorly aligned, and the anterior chamber remained in communication with the corneal surface, resulting in low pressure between corneal layers and adhesion. Four days after the operation, the corneal epithelium was repaired, and the external opening was closed, causing the aqueous humor to pour into the corneal stroma and opening the potential cavity due to an increase in interlayer pressure. Therefore, we propose that the pathogenesis of IFS is caused by both the gaping internal wound and the closed external wound. Both factors are indispensable, and the onset time depends on the closing time of the external opening of the perforating injury.

Treatment of IFS varies according to its different causes, including IOP lowering therapy and Descemet membrane endothelial keratoplasty. In this case, the treatment principle was to close the full-thickness wound in a timely manner. However, ensuring the suturing depth and contrapuntal alignment of the wound, especially the internal orifice, can be challenging due to edema and turbidity of the wound. Intraoperative optical coherence tomography (iOCT) enables real-time visualization of ocular structures during surgery and enhances our understanding of intraoperative dynamics. High-resolution OCT is now useful to aid more accurate measurements of cornea thickness, flap interface assessment, and minor flap displacements.

This case emphasizes that a history of corneal perforation after SMILE may be a potential causative factor of IFS. Additionally, it underscores the need to consider the full extent of any corneal wound during the repair process, with close attention paid to the suture depth, and iOCT plays a unique role in the operation.

## Data Availability

The datasets used during the current study are available from the corresponding author on reasonable request.
